# Clinical Outcomes of Liver Abscesses in Adults: A 10-Year Experience at a Tertiary Care Center in Northern India

**DOI:** 10.7759/cureus.75454

**Published:** 2024-12-10

**Authors:** Ayushya Gupta, Nancy Thakur, Ashish K Chaudhary, Udit Patel, Arti

**Affiliations:** 1 Department of General Surgery, Ganesh Shankar Vidyarthi Memorial Medical College, Kanpur, IND; 2 Department of Anatomy, Ganesh Shankar Vidyarthi Memorial Medical College, Kanpur, IND

**Keywords:** amoebic liver abscess, antimicrobial resistance, entamoeba histolytica, escherichia coli, hepatic abscess, india, klebsiella pneumonia, percutaneous catheter drainage, pyogenic liver abscess

## Abstract

Purpose

Hepatic abscesses remain a significant clinical challenge due to high morbidity and mortality. This research aims to examine the etiological spectrum, management approaches, clinical features, and results in hepatic abscesses in a tertiary care facility in northern India, emphasizing the distinctions among pyogenic liver abscesses (PLAs) and amoebic liver abscesses (ALAs).

Methods

This retrospective study was done at GSVM Medical College, Kanpur, analyzing 725 patients with hepatic abscesses over a 10-year period. Patients were included based on confirmed diagnoses of ALAs or PLAs through clinical, serological, and microbiological evidence. Data on demographics, clinical presentation, imaging findings, laboratory results, and management approaches were extracted from hospital records. IBM SPSS Statistics for Windows, Version 23 (Released 2015; IBM Corp., Armonk, New York, United States) was employed for statistical analysis, and continuous variables were displayed accordingly as means or medians and categorical variables as frequencies. Subgroup analyses were conducted based on abscess characteristics, including size, location, and etiology.

Results

Of the 725 patients analyzed, the mean age was 42.7 ± 15.8 years, with men comprising 85.93% of the cohort. ALAs accounted for 82.3% of cases, while PLAs comprised 12%. PLA cases frequently involved Gram-negative pathogens, such as Escherichia coli and Klebsiella pneumoniae, with 64% of PLA patients exhibiting positive pus or blood cultures. Common presenting symptoms included abdominal pain (87%) and fever (84.5%). Significant comorbidities included alcoholism (43.6%) and diabetes mellitus (34.2%). Right lobe involvement was predominant (75.9%), and multiple abscesses were noted in 47.5% of patients.

Complications included pleural effusion (53.9%), abscess rupture (16.3%), and systemic inflammatory response syndrome (25.7%). Management strategies comprised antibiotics, percutaneous catheter drainage (74.8%), and needle aspiration (43.4%), with conservative treatment being effective in smaller abscesses. Mortality rates were low, at 1.6% for ALAs and 1.5% for PLAs. Hospital stays were shorter for PLA cases (median: five days) compared to ALAs (median: 7.3 days).

Conclusion

This study underscores the predominance of ALAs in endemic regions like India and highlights the significant role of Gram-negative bacteria in PLAs. Tailored management strategies, including percutaneous interventions and early antibiotic therapy, were associated with favorable outcomes and low mortality. However, the emergence of antimicrobial resistance in PLAs warrants robust antimicrobial stewardship. Public health measures focused on sanitation and clean water are critical to reducing the prevalence of ALAs.

## Introduction

Liver abscesses (LAs) represent a significant clinical challenge, marked by localized infections in the hepatic parenchyma that can result in high morbidity and mortality. These abscesses are primarily classified as amoebic liver abscesses (ALAs) and pyogenic liver abscesses (PLAs), each differing in etiology, clinical presentation, and microbiological profiles [1,2]. Advances in diagnostic imaging, interventional radiology, and antibiotic therapies have revolutionized management strategies, yet substantial variability persists in treatment practices and outcomes [3,4].

In India, the most common type is an ALA because of the endemic presence of Entamoeba histolytica [5]. On the other hand, PLAs, often associated with biliary tract infections and systemic sepsis, exhibit a polymicrobial etiology, commonly involving organisms like Klebsiella pneumoniae and Escherichia coli [6,7]. Early detection, supported by imaging modalities such as ultrasonography and computed tomography, is pivotal to reducing complications like abscess rupture and systemic inflammatory responses [8,9].

Management strategies typically include antibiotic therapy and percutaneous interventions like needle aspiration or catheter drainage, based on the size of the abscess, location, and patient stability. Large abscesses (>10 cm) and bilobar presentations pose higher risks of complications and mortality [1,10,11].

In this study, we analyzed 725 cases of hepatic abscesses, focusing on clinical characteristics, therapeutic approaches, and patient outcomes. By examining diverse parameters such as presenting symptoms, abscess features, and intervention efficacy, the research aims to provide critical insights into optimizing management strategies for LA patients. We also propose a management algorithm for the treatment of patients with hepatic abscesses based on our experience.

## Materials and methods

This retrospective observational study was carried out at GSVM Medical College, Kanpur, analyzing the clinical characteristics and outcomes of hepatic abscesses in 725 patients in the last 10-year period from January 2014 to January 2024. The study utilized medical records to extract comprehensive data, focusing on demographic details, presenting symptoms, laboratory findings, imaging results, treatment modalities, and outcomes.

Study population

Patients included in the study were diagnosed with hepatic abscesses based on clinical evaluation, laboratory findings, and imaging studies, such as ultrasonography and computed tomography. The inclusion criteria comprised patients with confirmed LAs on imaging, while cases below the age of 18, with uncertain diagnoses or incomplete records, were excluded.

Data collection

Data were obtained from hospital records, which detailed patient demographics (age, gender), medical history (alcohol consumption, diabetes, liver cirrhosis), clinical presentation (fever, abdominal pain, jaundice), and laboratory testing (liver function tests, total blood count). Records of imaging studies were reviewed to document the abscess size, number, and location within the liver.

Classification and diagnosis

LAs were classified into amoebic or pyogenic based on clinical, microbiological, and serological evidence. ALAs were diagnosed through serological confirmation of Entamoeba histolytica or the characteristic “anchovy sauce” appearance on aspiration. PLAs were identified by positive bacterial cultures from blood or pus or associated biliary tract infections.

Management and intervention

In the absence of consensus guidelines, patients with LAs were managed as per the attending doctor. Patients were managed based on the severity and characteristics of their condition. In general, initial treatment involved metronidazole 1g eight hourly intravenously with injection ceftriaxone 1g 12 hourly intravenously as clinically indicated. Percutaneous interventions, including ultrasound-guided needle aspiration or catheter drainage, were performed for large or complicated abscesses, as well as cases refractory to medical therapy and pus sent for culture and sensitivity testing. Surgical intervention was reserved for abscesses with complications such as rupture or failure of minimally invasive procedures. Empirical therapy was revised based on culture sensitivity reports and sterile pus culture patients were continued on the same treatment. The treatment was continued for seven days intravenously then the patient was taken on oral medications in uncomplicated cases. Clinical improvement was characterized by the resolution of fever, normalization of leukocyte count, and alleviation of local signs and symptoms following successful percutaneous drainage (PCD) or percutaneous needle aspiration.

Objectives and outcome assessment

The objectives of the study were to analyze the etiological spectrum, clinical presentation, and management strategies for hepatic abscesses. A key objective was to compare the differences in clinical characteristics and outcomes between ALAs and PLAs. Specific outcome measures included symptom resolution (e.g., fever, abdominal pain), normalization of laboratory parameters (e.g., leukocyte count), imaging-confirmed reduction in abscess size, duration of hospital stay, and mortality rates. The study also aimed to evaluate the effectiveness of various treatment modalities, including antibiotics, percutaneous interventions (catheter drainage, needle aspiration), and surgical drainage, while assessing associated complications such as pleural effusion, abscess rupture, and systemic inflammatory response syndrome (SIRS). Based on these findings, the study proposes a tailored management algorithm, grounded in the institution’s 10-year experience, to guide evidence-based treatment of hepatic abscesses.

Statistical analysis

IBM SPSS Statistics for Windows, Version 23 (Released 2015; IBM Corp., Armonk, New York, United States) was applied to statistical evaluations. Continuous variables were shown as mean ± standard deviation (SD) or median + interquartile range (IQR), according to the data distribution, whilst categorical variables were shown as frequencies and percentages. Groups were compared using Mann-Whitney U tests for non-normally distributed data and independent t-tests for regularly distributed continuous variables. To assess variations in categorical variables, including complication rates, mortality, and gender distribution, Pearson's chi-square tests were used. Subgroup analyses were carried out to assess differences based on abscess characteristics such as size, location (right lobe, left lobe, bilobar), and underlying liver conditions like cirrhosis. For all tests, a p-value of less than 0.05 was deemed statistically important, ensuring a thorough assessment of the clinical and outcome parameters. The study data were accessible to all authors, who also examined and approved the final manuscript.

## Results

The study analyzed 725 patients with hepatic abscesses, with a mean age of 42.7 ± 15.8 years. Male patients predominated, accounting for 85.93% (623 patients) of the cohort. Baseline characteristics are tabulated in Table 1.

**Table 1:** Baseline Characteristics of the Studied Patients ^a^ Pyogenic liver abscess; ^b ^Amoebic liver abscess; SD, Standard deviation; IQR, Interquartile range; INR, International normalization ratio; Na, Sodium; K, Potassium.

Parameter	Values (N=725)
Age (years) (mean ± SD)	42.7 ± 15.8
Gender – male (%)	623 (85.93%)
Etiology, n (%)	
Pyogenic (PLA)^a^	87 (12%)
Amoebic (ALA)^b^	597 (82.3%)
Tubercular	4 (0.5%)
Fungal	1 (0.1%)
Post-liver transplant	2 (0.3%)
Eosinophilic	5 (0.7%)
Malignant	7 (0.1%)
Cryptogenic	22 (3 %)
Clinical presentation, n (%)	
Fever	613 (84.5%)
Abdominal pain	631 (87%)
Jaundice	72 (10%)
Loss of appetite	232 (32%)
Weight loss	105 (14.5%)
Shortness of breath	79 (10.9%)
Chest pain	44 (6.1%)
Abdominal lump	23 (3.2%)
Vomiting	104 (14.3%)
Cough	58 (8%)
Medical history, n (%)	
Alcoholism	316 (43.6%)
Diabetes mellitus	248 (34.2%)
Hypertension	83 (11.5%)
History of diarrhea	17 (2.3%)
History of blood in stools	4 (0.05%)
Gallstones	26 (3.6%)
Liver cirrhosis	41 (5.7%)
Examination, n (%)	
Pallor	271 (37.4%)
Pedal edema	81 (11.2%)
Lymphadenopathy	13 (1.8%)
Hepatomegaly	467 (64.4%)
Splenomegaly	146 (20.1%)
Laboratory parameters	
Plasma haemoglobin (g/L) [mean ± SD]	11.2 ± 2.3
Total leukocyte count (/mm³)	13000 (7100–19400)
Platelet count (×10⁹)	310 (202- 418)
Total bilirubin (mg/dL)	1.8 ± 0.9
Serum alanine aminotransaminases (IU/mL)	36 (26–58)
Serum alkaline phosphatase (IU/mL)	182 (120- 284)
Serum albumin (g/dL)	2.6 ± 0.7
INR	1.2 ± 0.54
Serum Na⁺ (mEq/L)	133.2 ± 5.6
Serum creatinine (mg/dL)	1.04 ± 0.64
Hepatitis B surface antigen positivity (%)	8 (1.1%)
Hepatitis C antibody positivity (%)	4 (0.5%)
Fasting blood sugar (mg/dL)	103 (91- 144)

The most frequent presenting symptoms were abdominal pain (87%) and fever (84.5%). Other symptoms included loss of appetite (32%), vomiting (14.3%), weight loss (14.5%), and jaundice (10%). Shortness of breath, chest pain, abdominal lump, and cough were less commonly reported. Comorbidities were prevalent, with 43.6% of patients reporting chronic alcoholism, 34.2% having diabetes mellitus, and 5.7% presenting with liver cirrhosis. On physical examination, hepatomegaly was noted in 64.4% of patients, followed by pallor (37.4%) and splenomegaly (20.1%).

Laboratory investigations revealed a mean hemoglobin level of 11.2 ± 2.3 g/L and a median leukocyte count of 13,000/mm³ (IQR: 7,100-19,400). Platelet counts averaged 310 × 10⁹/L, & the mean serum albumin level is 2.6 ± 0.7 g/dL, indicative of hypoalbuminemia in many cases. Total bilirubin levels averaged 1.8 ± 0.9 mg/dL, while serum alkaline phosphatase (ALP) and alanine aminotransferase (ALT) median values are 36 IU/mL (IQR: 26-58) and 182 IU/mL (IQR: 120-284), respectively. Electrolyte disturbances were reflected in a mean serum sodium level of 133.2 ± 5.6 mEq/L. The fasting blood sugar levels had a median value of 103 mg/dL (IQR: 91-144).

**Figure 1:**
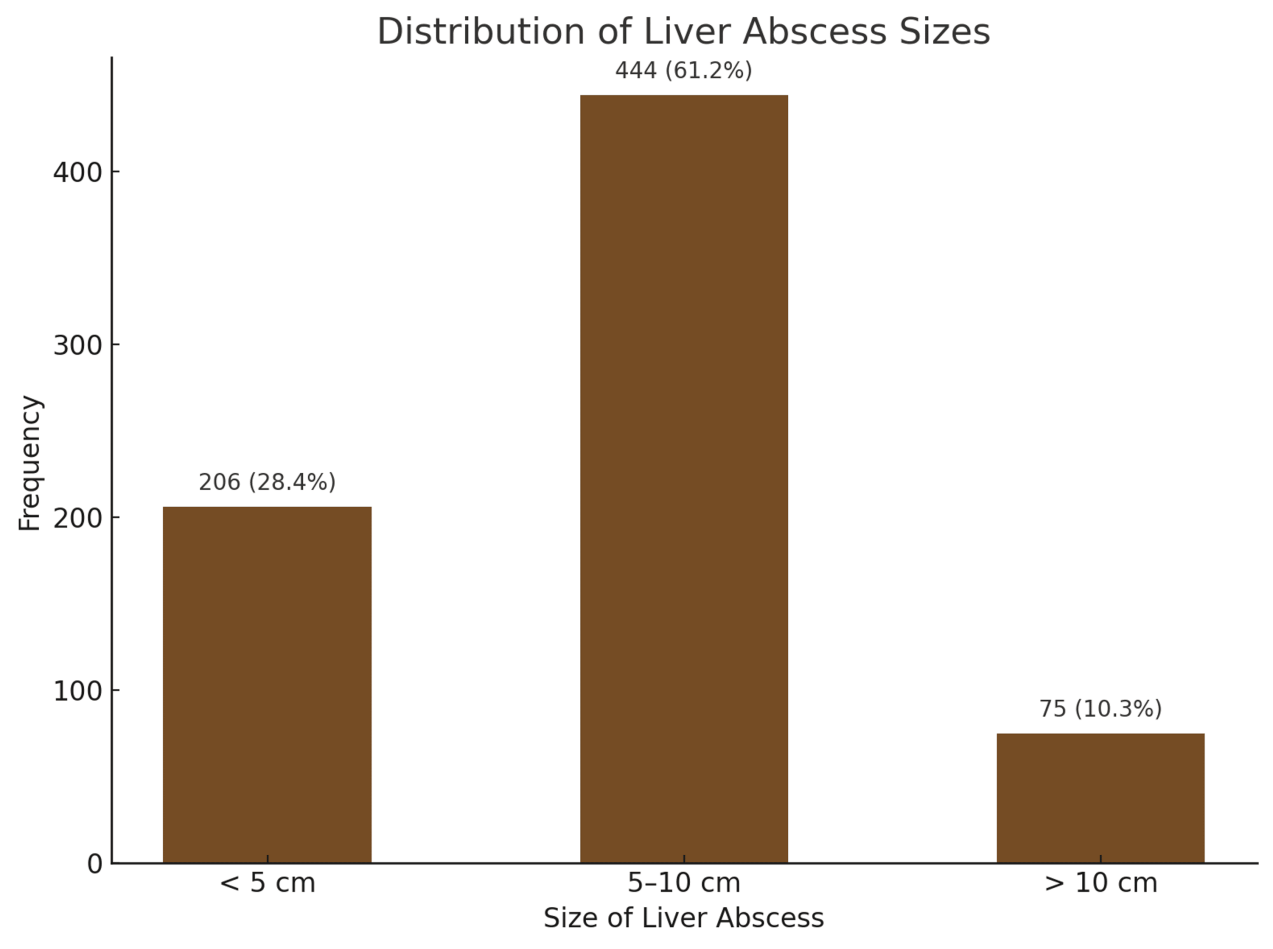
Distribution of Liver Abscess Sizes

The majority of liver abscesses (61.2%) measured between 5 and 10 cm in size. Smaller abscesses, measuring less than 5 cm, were observed in 28.4% of cases, while larger abscesses, exceeding 10 cm, were the least frequent at 10.3% (Figure 1).

**Figure 2:**
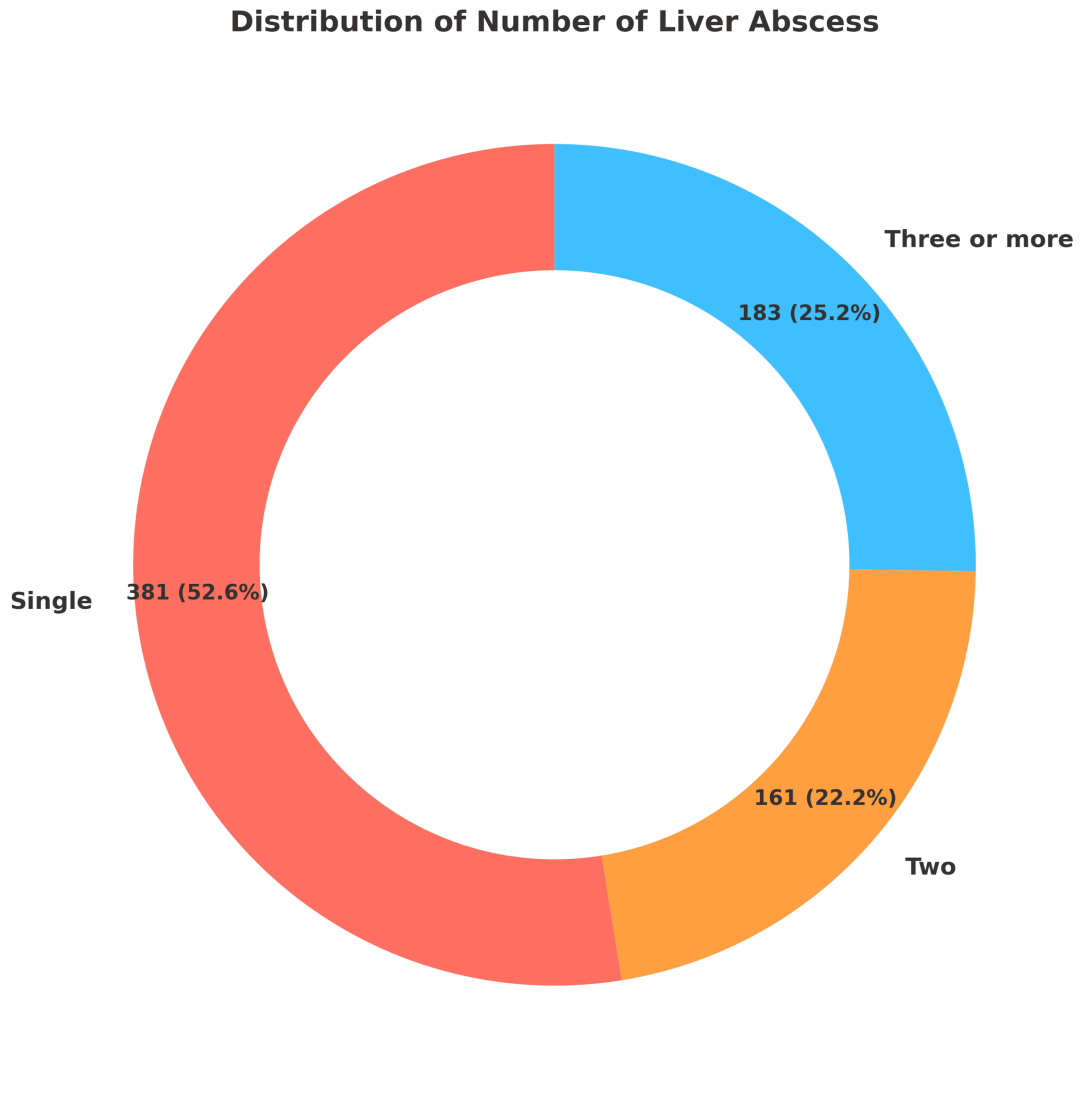
Distribution of the Number of Liver Abscesses

In terms of abscess characteristics, 52.6% of patients had a single abscess, while 47.4% of patients had more than one abscess. 22.2% had two abscesses and 25.2% presented with three or more (Figure 2).

The right lobe was the most commonly involved site (75.9%), followed by the left lobe (14.2%), and bilobar involvement in 9.9% of cases (Table 2). Subcapsular abscesses were identified in 44.4%, and 3.3% were subdiaphragmatic. The mean maximum diameter of the abscesses was 7.06 ± 2.82 cm, with 10.3% measuring larger than 10 cm. The median abscess volume was 152 mm³ (IQR: 59-345) (Table 2).

**Table 2:** Liver Abscess Characteristics: Number, Location, and Size

Parameter	Values (N=725)
Number of lesions, n (%)	
Single	381 (52.6%)
Multiple	344 (47.5%)
Two	161 (22.2%)
Three or more	183 (25.2%)
Location of lesions, n (%)	
Right lobe	550 (75.9%)
Left lobe	103 (14.2%)
Right + left lobe	72 (9.9%)
Segment I	40 (5.5%)
II	67 (9.3%)
III	66 (9.1%)
IV	119 (16.4%)
V	107 (14.8%)
VI	191 (26.3%)
VII	284 (39.2%)
VIII	216 (29.8%)
Subcapsular	322 (44.4%)
Subdiaphragmatic	24 (3.3%)
Size of lesions, n (%)	
< 5 cm	206 (28.4%)
5–10 cm	444 (61.3%)
> 10 cm	75 (10.3%)
Mean max. diameter (cm)	7.06 ± 2.82
Median volume (cu. mm)	152 (59–345)

This study revealed a diverse etiological spectrum of LAs, with ALAs being the most common, accounting for 82.3% of cases (n=597), followed by PLAs, which comprised 12% (n=87). Among the PLA cases, 87 patients had positive pus cultures, and 64 had positive blood cultures (Table 3).

**Table 3:** Organism Isolated in Pyogenic Liver Abscesses CRK, Carbapenem-resistant Klebsiella; VRE, Vancomycin-resistant Enterococcus

Organism isolated	Pus (N =87)	Blood (N= 64)
Gram (-) bacteria, n		
Escherichia coli	44	27
Klebsiella pneumoniae	20 (2 CRK)	15
Pseudomonas	7	6
Burkholdaria sp.	2	1
Acinetobacter	3	3
Gram (+) bacteria, n		
Staphylococcus aureus	5	2
Enterococcus faecium	3	6 (1 VRE)
CONS	3	3
Fungal, n	0	1

**Figure 3:**
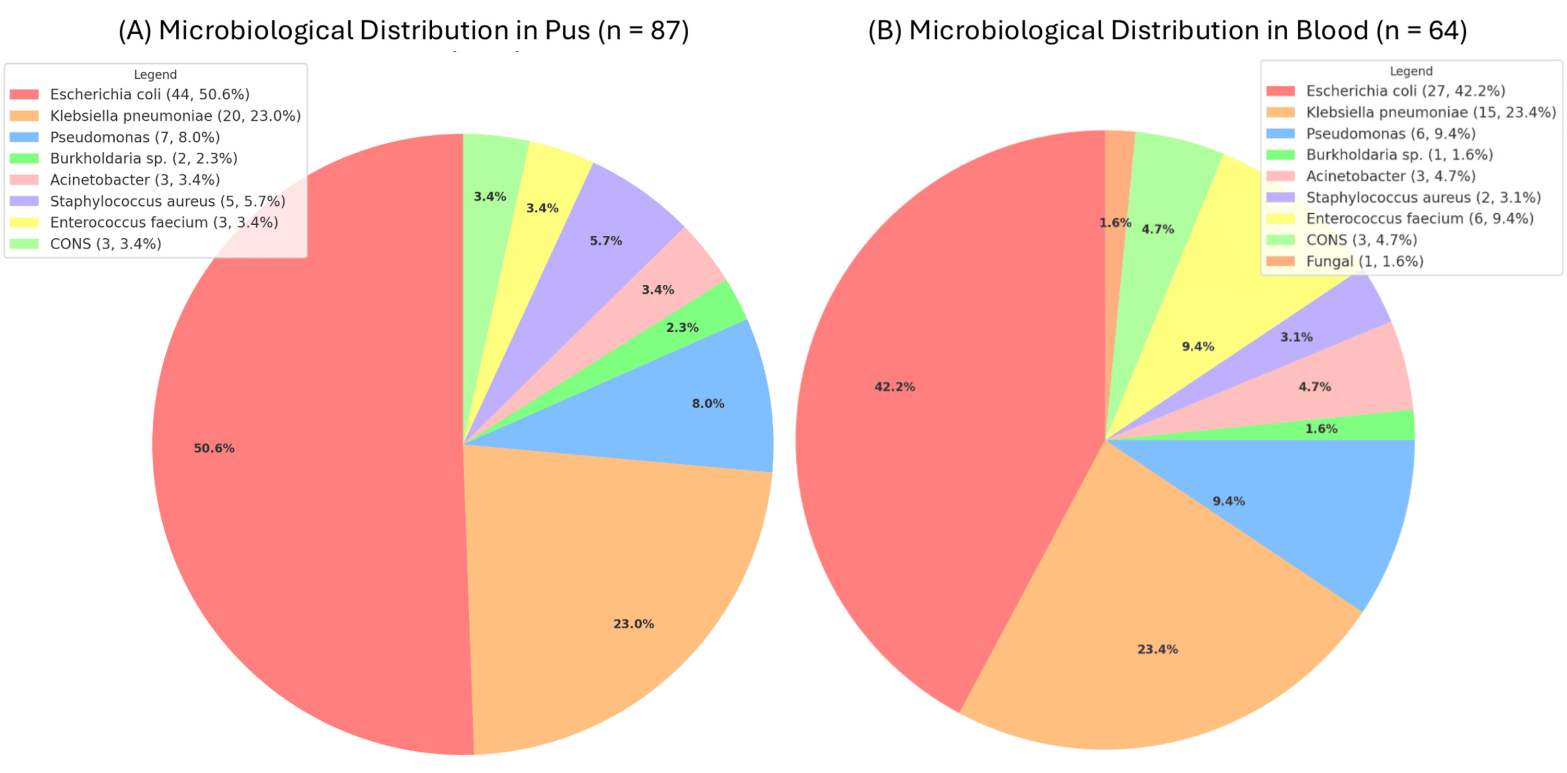
Isolated Organisms in Pyogenic Liver Abscess: (A) Microbiological Distribution in Pus and (B) Microbiological Distribution in Blood CONS, Coagulase-negative staphylococci

The predominant pathogens isolated from Gram-negative bacteria, which include Escherichia coli (n = 44) and Klebsiella pneumoniae (n = 20), were found in pus cultures and blood cultures showed similar results (E. coli, n = 27; K. pneumoniae, n = 15). Additional Gram-negative bacteria found in pus and blood cultures included Pseudomonas (n=7 and n=6, respectively), Burkholderia species (n=2 and n=1, respectively), and Acinetobacter (n=3 in both pus and blood cultures). Gram-positive bacteria were less frequently isolated, with Staphylococcus aureus identified in five pus samples and two blood samples. Enterococcus faecium is the most prevalent organism that was Gram-positive in blood cultures (n=6, including one vancomycin-resistant strain), while it was isolated in three pus cultures. Coagulase-negative staphylococci (CONS) were identified in three pus and three blood cultures. Fungal infections were rare, with a single fungal isolate detected in blood cultures (Figure 3).

Other rare etiologies included eosinophilic abscesses (0.7%, n=5), characterized by Charcot-Leyden crystals in pus cultures, tubercular abscesses (0.5%, n=4), and post-liver transplant abscesses (0.3%, n=2). Malignant abscesses accounted for 0.1% (n=7), with some associated with biliary malignancies. Cryptogenic abscesses accounted for 3%.

Complications were common among the cohort (Table 4).

**Table 4:** Complications of Liver Abscesses SIRS, Systemic inflammatory response syndrome

Parameter	Values (N=725)
Local complications, n (%)	
Pleural effusion	391 (53.9%)
Abscess rupture	118 (16.3%)
Biliary rupture	3 (0.4%)
Intraperitoneal rupture	73 (10%)
Intrapleural rupture	42 (5.8%)
Systemic complications, n (%)	
Pneumonia	73 (10.1%)
Urosepsis	41 (5.7%)
SIRS	186 (25.7%)
Encephalopathy	0 (0%)
Acute kidney injury	108 (14.9%)

The most common local complication, affecting 53.9% of patients, was pleural effusion. Abscess rupture occurred in 16.3%, with intraperitoneal rupture being the most frequent (10%), followed by intrapleural rupture (5.8%) and biliary rupture (0.4%). Systemic complications included systemic inflammatory response syndrome (SIRS) in 25.7% and acute kidney injury in 14.9% of patients. Pneumonia was observed in 10.1%, and urosepsis was noted in 5.7%. Notably, encephalopathy was absent in this cohort, suggesting limited progression to severe systemic involvement.

A comparison between ALAs and PLAs revealed notable differences in demographic, clinical, and biochemical profiles (Table 5).

**Table 5:** Comparison of Amoebic Liver Abscess (ALA) Versus Pyogenic Liver Abscess (PLA) SD, Standard deviation; IQR, interquartile range; Na, sodium; K, potassium; SIRS, systemic inflammatory response syndrome; PCD, percutaneous drainage; INR,  international normalization ratio *P-value<0.05 is significant. An independent t-test is done for age, plasma hemoglobin, serum albumin, and median diameter. The Mann-Whitney U test was applied for duration of fever, duration of abdominal pain, total leukocyte count, total bilirubin, serum alanine aminotransaminases, serum aspartame aminotransaminases, serum alkaline phosphatase, INR, median volume, duration of PCD and duration of hospitalization. For all other parameters, Pearson’s Chi-square test was used.

Parameter	Amoebic (ALA) (n = 597)	Pyogenic (PLA) (n = 87)	p-value
Age (years) (mean ± SD)	42.9	49.7	<0.001*
Gender – male (%)	511 (85.5%)	67 (77.0%)	0.056
Clinical and lab parameters			
Fever – n (%)	512 (85.8%)	74 (84.7%)	0.001*
Duration of fever (days) (median, IQR)	10 (7–15)	12 (8–18)	0.001*
Abdominal pain – n (%)	519 (86.9%)	70 (80.8%)	0.14
Duration of abdominal pain (days) (median, IQR)	10 (7–16)	12 (8–18)	0.001*
Jaundice – n (%)	53 (9.0%)	22 (24.7%)	0.001*
Shortness of breath – n (%)	85 (14.3%)	9 (9.9%)	0.412
Chest pain – n (%)	46 (7.7%)	14 (16.1%)	0.017*
Alcoholism – n (%)	211 (35.3%)	38 (43.7%)	0.164
Diabetes mellitus – n (%)	215 (35.9%)	24 (27.4%)	0.155
Gallstones – n (%)	2 (0.4%)	7 (8.1%)	0.001*
Hepatomegaly – n (%)	390 (65.4%)	42 (48.3%)	0.003*
Splenomegaly – n (%)	137 (23.0%)	16 (18.6%)	0.414
Plasma hemoglobin (g/L) [mean ± SD]	8.7	11	0.001*
Total leukocyte count (/mm³)	13,100 (3,200–26,400)	12,800 (3,000–24,000)	0.302
Total bilirubin (mg/dL)	1.9	4.2	0.001*
Serum alanine aminotransaminases (IU/mL)	36 (25–57)	42 (26–68)	0.363
Serum aspartate aminotransaminases (IU/mL)	37 (24–60)	33 (18–59)	0.221
Serum alkaline phosphatase (IU/mL)	182 (120–284)	201 (135–315)	0.002*
Serum albumin (g/dL)	2.5 ± 0.7	2.6 ± 0.8	0.148
INR	1.2 ± 0.5	1.3 ± 0.4	0.007*
Complications–n (%)			
Pleural effusion – n (%)	312 (52.3%)	48 (55.0%)	0.694
Abscess rupture – n (%)	78 (13.1%)	22 (25.1%)	0.004*
Biliary rupture – n (%)	19 (3.1%)	7 (7.6%)	0.055
Intraperitoneal rupture – n (%)	60 (10.1%)	10 (11.7%)	0.821
Intrapleural rupture – n (%)	40 (6.6%)	3 (4.0%)	0.351
Pneumonia – n (%)	68 (11.4%)	12 (13.8%)	0.636
SIRS – n (%)	123 (20.5%)	28 (31.7%)	0.021*
Encephalopathy – n (%)	14 (2.3%)	1 (1.6%)	0.749
Acute kidney injury – n (%)	89 (14.9%)	13 (15.0%)	1
Abscess characteristics			
Number (single %)	326 (54.6%)	51 (58.4%)	0.556
Location (Right lobe %)	462 (77.5%)	67 (77.1%)	1
Bilobar abscess (%)	29 (4.8%)	13 (14.4%)	0.001*
Sub capsular (%)	270 (45.2%)	36 (41.5%)	0.571
Subdiaphragmatic (%)	7 (1.3%)	5 (5.8%)	0.009*
Size <5 cm (%)	150 (25.1%)	25 (28.6%)	0.555
Size >10 cm (%)	61 (10.3%)	11 (12.3%)	0.615
Median diameter (cm)	7.02 ± 2.95	7.16 ± 2.81	0.013*
Median volume (cu. mm)	135 (67–318)	165 (60–377)	0.001*
Management and outcomes			
Conservative (%)	111 (18.5%)	17 (19.5%)	0.948
Interventions (%)	486 (81.5%)	70 (80.5%)	0.948
Aspiration (single + multiple) (%)	207(34.7%)	21 (24.1%)	0.067
PCD (%)	275 (46.1%)	47(54%)	0.202
Surgical Drainage (%)	4 (0.7%)	2 (2.3%)	0.364
Duration of PCD (days)	15 (10–20)	10 (7–19)	0.001*
Duration of hospitalization (days)	7.3 (5.3–12.4)	5 (4–7.1)	0.001*
Outcome (mortality %)	10 (1.6%)	1 (1.5%)	0.815

Patients with ALAs were generally younger, with a mean age of 42.9 years, compared to 49.7 years in PLA cases (p = 0.029). Male predominance was more pronounced in ALA (85.5%) than in PLA (77%; p = 0.005). Fever was slightly more frequent in ALA cases (85.8% vs. 84.7%; p = 0.001), as was abdominal pain (86.9% vs. 80.8%; p = 0.001. On the other hand, jaundice was seen in 24.7% of PLA cases, compared to 9% of ALA cases (p = 0.001). In PLA, chest pain was also more common. (16.1% vs. 7.7%; p = 0.001).

Comorbidities also differed between the two groups. Alcoholism was more prevalent in PLA patients (43.7% vs. 35.3%; p = 0.012), while diabetes mellitus was more common in ALAs (35.9% vs. 27.4%; p = 0.041). Gallstones were significantly associated with PLAs (8.1%) compared to ALA (0.4%; p = 0.015). In terms of laboratory parameters, PLA cases had higher total bilirubin levels (4.2 mg/dL vs. 1.9 mg/dL; p = 0.001) and elevated alkaline phosphatase levels (201 IU/mL vs. 182 IU/mL; p = 0.002). Abscess rupture was more frequent in PLA (25.1%) than in ALA (13.1%; p = 0.001), and SIRS was significantly higher in PLA patients (31.7% vs. 20.5%; p = 0.001).

Management of hepatic abscesses included conservative treatment with antibiotics or procedural interventions like PCD, needle aspiration, or surgical drainage. Conservative management was successful in 18.5% of ALA cases and 19.5% of PLA cases (p = 0.948). Most patients required interventions, with PCD being the most common, performed in 46.1% of ALA cases and 54% of PLA cases. Needle aspiration was more frequently used in ALA (34.7% vs. 24.1%; p = 0.067). The duration of PCD was significantly shorter in PLA patients (10 days vs. 15 days; p = 0.001). Surgical drainage was needed in four patients (0.7%) with ALAs and two patients (2.3%) with PLAs. Early intervention contributed to better outcomes, with the majority of complications, such as pleural effusion and abscess rupture, managed effectively.

The hospitalization duration was also shorter for PLA cases (5 days vs. 7.3 days; p = 0.001), reflecting a potentially more favorable response to interventions. Despite the differences in clinical presentation and management, mortality rates were low and comparable among the two groups (1.6% in ALA vs. 1.5% in PLA; p = 0.815).

## Discussion

This study provides a comprehensive examination of the etiological spectrum, clinical features, treatment, as well as outcomes of hepatic abscesses. The findings reaffirm the predominance of ALAs in this region, accounting for 82.3% of cases, while PLAs constituted 12%. This distribution is consistent with studies from other regions in India, where ALAs are prevalent due to poor sanitation and widespread exposure to Entamoeba histolytica [1,5]. Similar trends have been observed in other endemic regions, emphasizing the importance of environmental and hygienic factors [12]. The male predominance (85.93%) and younger age profile in ALA cases align with previous research, which attributes these trends to occupational and lifestyle factors, including higher alcohol consumption among men [13,14].

The etiological findings for PLAs in this study are particularly noteworthy. The most prevalent pathogens are gram-negative bacteria, Klebsiella pneumoniae and Escherichia coli being predominant, as reported in similar studies [6,7]. The detection of carbapenem-resistant Klebsiella strains underscores the increasing challenge of antimicrobial resistance, although successful treatment with colistin highlights the importance of timely and appropriate antibiotic selection. Other studies have also reported the emergence of multidrug-resistant Klebsiella pneumoniae in PLAs, emphasizing the need for global antimicrobial resistance vigilance [15]. Polymicrobial infections, observed in eight cases, and the association with biliary diseases such as choledocholithiasis and biliary malignancies, align with findings in Western studies that highlight the role of biliary tract pathology in PLA etiology [16,17]. The identification of eosinophilic abscesses with Charcot-Leyden crystals and the rare occurrence of fungal and tubercular abscesses add to the understanding of the diverse etiological spectrum of LAs.

The clinical presentation of hepatic abscesses in this study was dominated by fever (84.5%) and abdominal pain (87%), consistent with prior studies [8,10]. Jaundice, present in 10% of cases, was more common in PLAs, reflecting its association with biliary pathology and systemic inflammation [5,9]. Laboratory findings showed leukocytosis, hypoalbuminemia, and elevated liver enzymes, all markers of systemic infection and hepatic involvement reported in previous studies [1,2,18].

The present research highlights the efficacy of needle aspiration and PCD, which were utilized in most cases of large or refractory abscesses. The preference for PCD is supported by prior studies demonstrating its superiority in larger abscesses and cases with multiloculated collections [19,20]. The median duration of PCD was shorter in PLA cases, likely due to faster resolution with targeted antibiotic therapy. Conservative management with antibiotics alone was successful in smaller abscesses, highlighting the importance of individualized treatment based on abscess size, location, and etiology [9,21]. The low mortality rate (1.6% in ALAs and 1.5% in PLAs) reflects improvements in early diagnosis and the use of minimally invasive techniques, consistent with global trends [13,22].

The predominance of right lobe involvement (75.9%) observed in this study aligns with anatomical studies explaining the preferential blood flow to the right lobe [5]. The association of PLAs with biliary diseases, including choledocholithiasis and biliary malignancies, mirrors findings from studies in Western populations [16,17]. The detection of carbapenem-resistant Klebsiella pneumoniae in PLA cases is consistent with recent studies reporting antimicrobial resistance as a growing concern [15]. The eosinophilic abscess cases identified here are rare and emphasize the importance of considering this etiology in patients with unexplained abscesses and negative cultures, as previously described [9].

This study has several important clinical implications. The high prevalence of ALAs underscores the need for public health interventions focused on sanitation and the prevention of Entamoeba histolytica infection. Health education campaigns and access to clean drinking water are critical in reducing the burden of amoebic abscesses. For PLAs, the findings emphasize how crucial early detection and treatment of biliary diseases is, which were a significant contributor to its etiology.

The majority of the time, percutaneous procedures are successful underscores the value of minimally invasive techniques in managing hepatic abscesses, which reduce hospital stays and complications. Early imaging and culture-guided antibiotics are critical. Training programs for PD could reduce the need for surgical interventions. Standardized protocols for referral and management may improve outcomes. Additionally, the emergence of carbapenem-resistant pathogens emphasizes the need for robust antimicrobial stewardship programs and the availability of advanced antibiotics for treating resistant infections.

**Figure 4:**
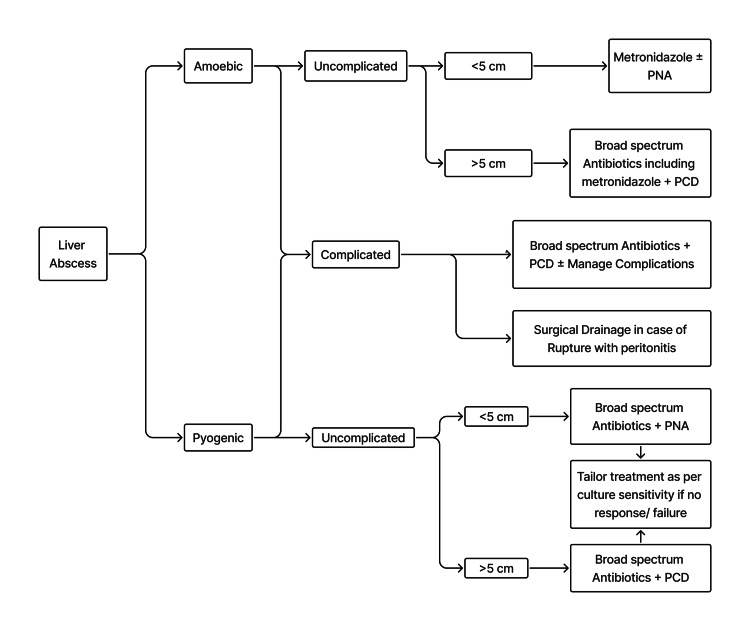
Algorithm to Treat Patients With Liver Abscesses Based on Experience at Our Center PNA, Percutaneous needle aspiration; PCD, Percutaneous catheter drainage.

Based on our experience, we suggest a treatment algorithm for managing LAs, though it still requires prospective validation (Figure 4).

Limitations

The study’s strengths include its large sample size of 725 patients, which provides robust statistical power and insights into the clinical spectrum of hepatic abscesses over a decade. Additionally, it uniquely compares ALAs and PLAs, offering comprehensive data on demographic, clinical, and treatment outcomes, culminating in a practical, experience-based management algorithm tailored to resource-limited settings.

However, there are several restrictions on this study. Being a single-center, retrospective study, it is susceptible to selection and documentation biases, and its findings may not be generalizable to other regions with different etiological patterns or healthcare resources. The absence of long-term follow-up data limits the ability to evaluate recurrence rates and the long-term effectiveness of management strategies. Moreover, while microbiological data were presented, our study did not include detailed antibiotic susceptibility profiles for all isolates, which could have provided deeper insights into resistance trends. Prospective, multicenter studies with long-term follow-up along with detailed microbiological analyses are needed to address these limitations and further enhance our understanding of hepatic abscesses.

## Conclusions

This study highlights the significant burden of hepatic abscesses, with ALAs being the most common etiology in this region. The findings emphasize the importance of early diagnosis and tailored management strategies, including the effective use of antibiotics and percutaneous interventions such as catheter drainage. The low mortality rates observed reflect advancements in minimally invasive techniques and supportive care. However, the emergence of antimicrobial resistance in PLAs underscores the need for robust antimicrobial stewardship. Improved public health measures, including better sanitation and access to clean water, are essential to reduce the prevalence of ALAs. Future studies should focus on long-term outcomes and strategies to address the challenges posed by resistant pathogens.

## References

[1] Jindal A, Pandey A, Sharma MK (2021). Management practices and predictors of outcome of liver abscess in adults: a series of 1630 patients from a liver unit. J Clin Exp Hepatol.

[2] Ghosh S, Sharma S, Gadpayle AK, Gupta HK, Mahajan RK, Sahoo R, Kumar N (2014). Clinical, laboratory, and management profile in patients of liver abscess from northern India. J Trop Med.

[3] Haque R, Huston CD, Hughes M, Houpt E, Petri WA Jr (2003). Amebiasis. N Engl J Med.

[4] Roediger R, Lisker-Melman M (2020). Pyogenic and amebic infections of the liver. Gastroenterol Clin North Am.

[5] Serraino C, Elia C, Bracco C (2018). Characteristics and management of pyogenic liver abscess: a European experience. Medicine (Baltimore).

[6] Rahimian J, Wilson T, Oram V, Holzman RS (2004). Pyogenic liver abscess: recent trends in etiology and mortality. Clin Infect Dis.

[7] Meddings L, Myers RP, Hubbard J (2010). A population-based study of pyogenic liver abscesses in the United States: incidence, mortality, and temporal trends. Am J Gastroenterol.

[8] Singh V, Bhalla A, Sharma N, Mahi SK, Lal A, Singh P (2008). Pathophysiology of jaundice in amoebic liver abscess. Am J Trop Med Hyg.

[9] Johannsen EC, Sifri CD, Madoff LC (2000). Pyogenic liver abscess. Infect Dis Clin North Am.

[10] Abbas MT, Khan FY, Muhsin SA, Al-Dehwe B, Abukamar M, Elzouki AN (2014). Epidemiology, clinical features and outcome of liver abscess: a single reference center experience in Qatar. Oman Med J.

[11] Huang CJ, Pitt HA, Lipsett PA, Osterman FA Jr, Lillemoe KD, Cameron JL, Zuidema GD (1996). Pyogenic hepatic abscess. Changing trends over 42 years. Ann Surg.

[12] Pérez JA, González JJ, Baldonedo RF (2001). Clinical course, treatment, and multivariate analysis of risk factors for pyogenic liver abscess. Am J Surg.

[13] Khim G, Em S, Mo S, Townell N (2019). Liver abscess: diagnostic and management issues found in the low resource setting. Br Med Bull.

[14] Sreeramulu PN, Swamy SD, Suresh V, Suma S (2019). Liver abscess: presentation and an assessment of the outcome with various treatment modalities. Int Surg J.

[15] Seewald S, Ang TL, Teng KC (2005). EUS-guided drainage of hepatic abscess. Endoscopy.

[16] Kaplan GG, Gregson DB, Laupland KB (2004). Population-based study of the epidemiology of and the risk factors for pyogenic liver abscess. Clin Gastroenterol Hepatol.

[17] Sharma MP, Ahuja V (2003). Amoebic liver abscess. J Indian Acad Clin Med.

[18] Chan KS, Chen CM, Cheng KC, Hou CC, Lin HJ, Yu WL (2005). Pyogenic liver abscess: a retrospective analysis of 107 patients during a 3-year period. Jpn J Infect Dis.

[19] Mohsen AH, Green ST, Read RC, McKendrick MW (2002). Liver abscess in adults: ten years experience in a UK centre. QJM.

[20] Haque R, Mondal D, Duggal P (2006). Entamoeba histolytica infection in children and protection from subsequent amebiasis. Infect Immun.

[21] Bächler P, Baladron MJ, Menias C (2016). Multimodality imaging of liver infections: differential diagnosis and potential pitfalls. Radiographics.

[22] Mukhopadhyay M, Saha AK, Sarkar A, Mukherjee S (2010). Amoebic liver abscess: presentation and complications. Indian J Surg.

